# Correction: Temporal gating of nuclear import: How Merkel cell polyomavirus exploits the cell cycle for nuclear entry

**DOI:** 10.1371/journal.ppat.1014095

**Published:** 2026-04-02

**Authors:** Karen Wang, Adrienne N. Eady, Isabel Amaya, Alina Stanczak, Chelsey C. Spriggs

The graph in Fig 5F of this article [1] is incorrect and the description of Fig 5G is missing from the figure legend. An updated Fig 5 with the correct graph and an updated figure legend is provided with this notice. The updated figure was assessed by members of the *PLOS Pathogens* Editorial Board who confirmed that the article’s overall findings are unaffected by this error.

In addition, the individual-level data and raw FACS data underlying Figs 1-5, and S2 were not originally provided with this article. The authors have provided these data as S1 File and S2 File.

With this Correction, all relevant data are provided in the below Supporting Information files.

**Fig 5:**
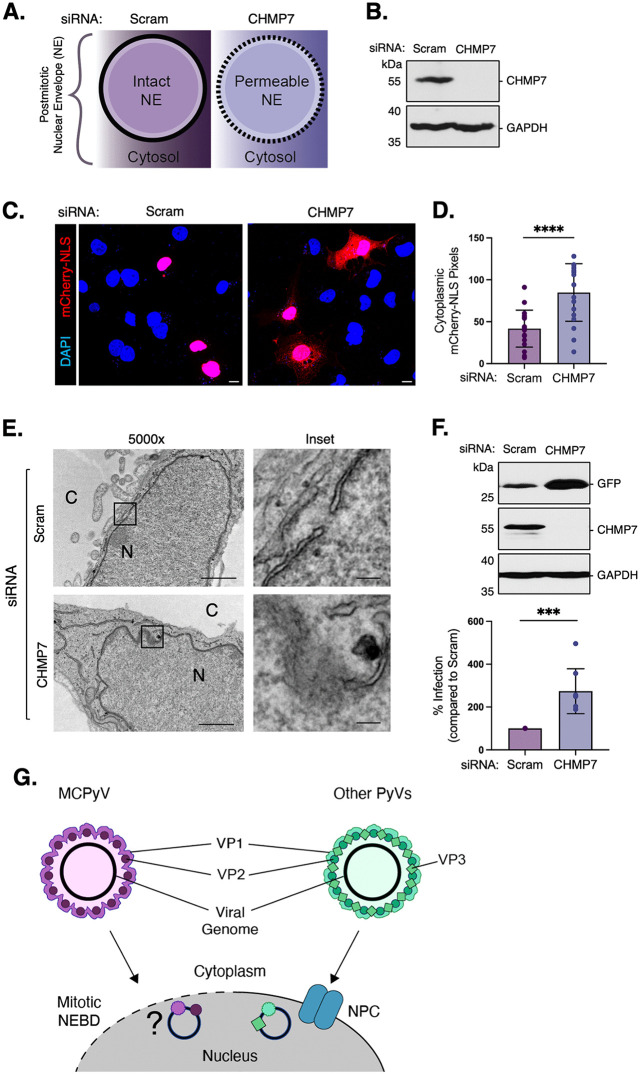
Increasing membrane permeability is sufficient to support MCPyV nuclear entry. **(A)** Schematic illustrating CHMP7’s role in nuclear envelope reformation. **(B)** COS-7 cells were transfected with 50 nM of either scrambled control siRNA (Scram) or siRNA against CHMP7. CHMP7 protein levels were assessed by immunoblotting and GAPDH was used as a loading control. **(C)** Confocal microscopy of COS-7 cells that were transfected with 50 nM of either scrambled control siRNA (Scram) or siRNA against CHMP7 for 24 h and then mCherry-NLS for an additional 24 h. Cells were then fixed and counterstained with DAPI (blue). Scale bars: 10 μm. **(D)** Quantification of (C) was performed using EBImage in R with adaptive thresholding (n  =  3, 50 cells/replicate). **(E)** TEM images of nuclear envelope morphology in COS-7 cells that were transfected with either scrambled control siRNA (Scram) or siRNA against CHMP7. Scale bars: 1000 nm (left), 100 nm (inset). **(F)** As in **(B)**, except cells were infected with MCPyV-GFP for 48 h. GFP and CHMP7 protein levels were assessed by immunoblotting and GAPDH was used as a loading control. **(G)** Model of MCPyV nuclear entry (left) compared to other well-studied PyVs (right). Values represent means  ±  SD from at least three independent experiments normalized to the loading control. Statistical significance was determined using an unpaired two-tailed Student’s t-test (***p  ≤  0.001, **** p  ≤  0.0001).

## Supporting information

S1 File Individual level data underlying graphs presented in Figs 1–5, and S2. (XLSX)

S2 File Raw FACS files underlying the results published in Figs 4 and S2. (ZIP)
