# Supplementary figures and images for: Temporal gating of nuclear import: How Merkel cell polyomavirus exploits the cell cycle for nuclear entry
Source: PLoS Pathog. 2025 May 30;21(5):e1013217. doi: 10.1371/journal.ppat.1013217 (PMC12157840; doi:10.1371/journal.ppat.1013217)

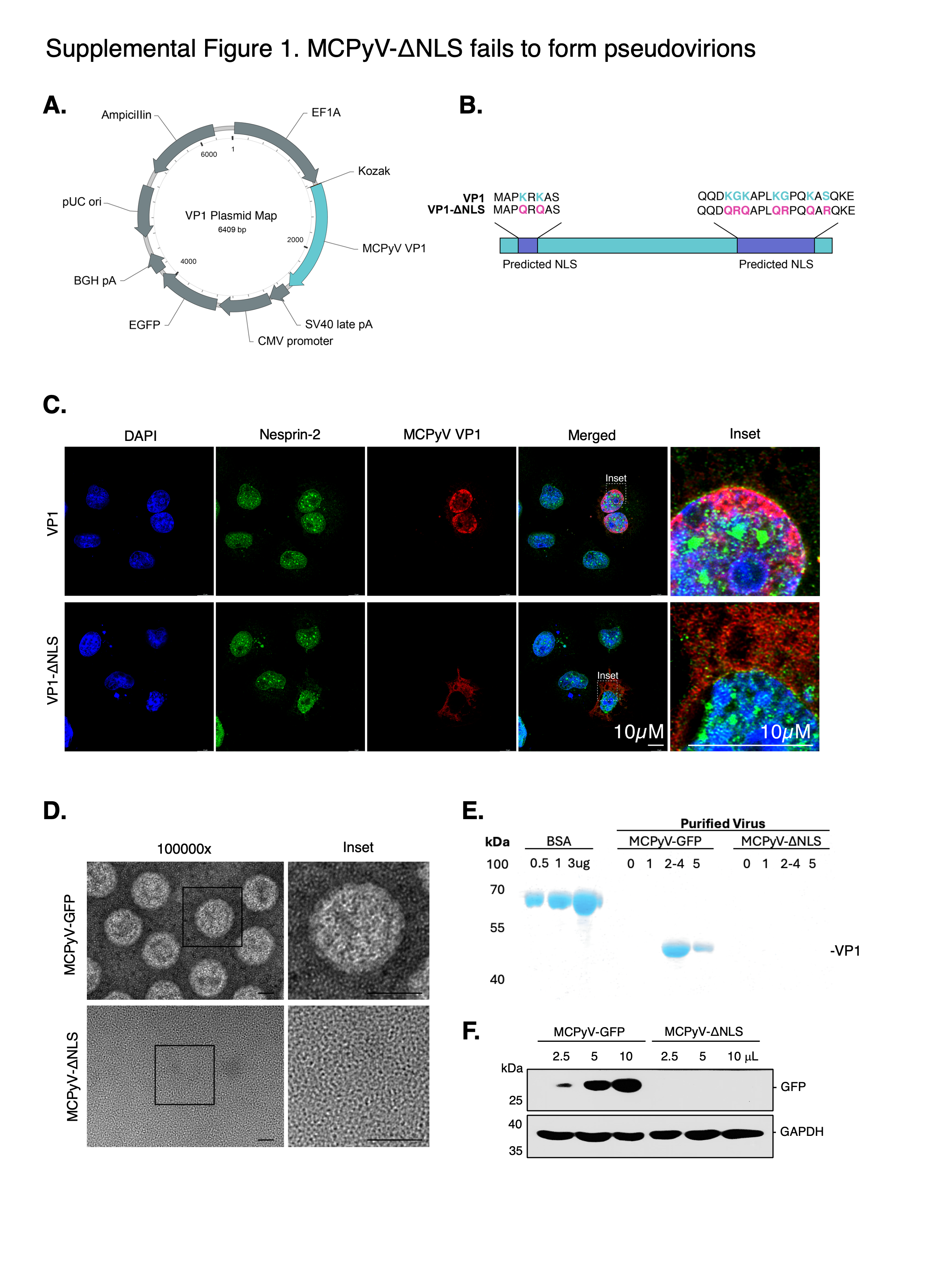

Supplement: S1 Fig — (A) Plasmid map of the VP1 expression construct. (B) Amino acid sequences of VP1 (wild-type) and VP1-ΔNLS, showing the mutations that were introduced in the predicted nuclear localization signal (NLS). (C) Immunofluorescence microscopy of cells transfected with VP1 or VP1-ΔNLS constructs. Scale bars: 10μm. (D) Transmission electron microscopy (TEM) images of purified MCPyV-GFP and MCPyV-ΔNLS preparations, showing normal pseudovirion formation with wild-type VP1 but not VP1-ΔNLS. Scale bars: 20 nm. (E) Coomassie-stained gel of purified virus preparations demonstrating the presence of VP1 protein in MCPyV-GFP but not MCPyV-ΔNLS samples. (F) COS-7 cells infected with equal amounts of MCPyV-GFP or MCPyV-ΔNLS pseodvirus from fractions 2–4 for 72 h. GFP expression indicates successful infection. (TIFF) [file ppat.1013217.s001.tiff]

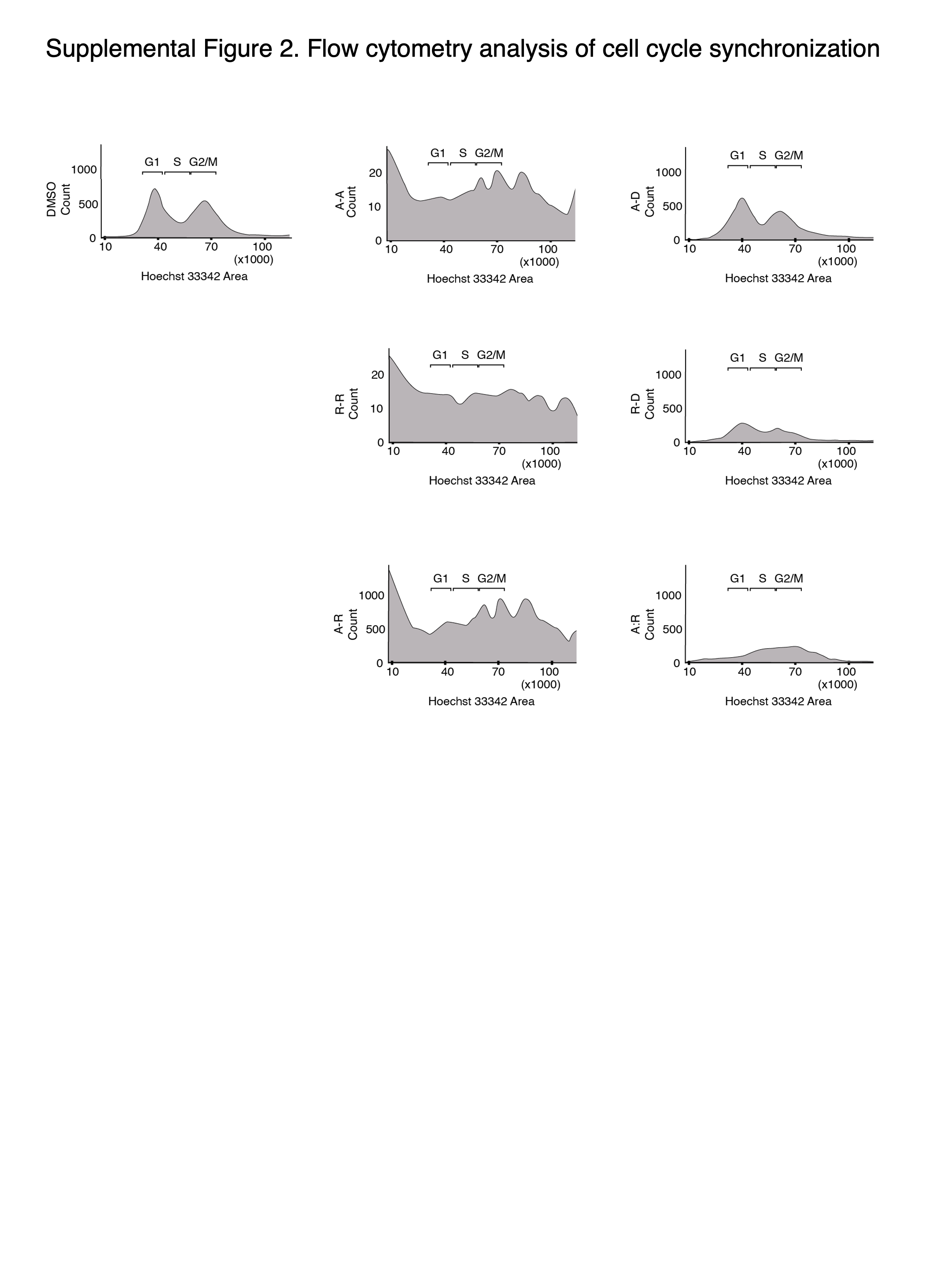

Supplement: S2 Fig — Flow cytometry analysis of Hoechst-labeled cells confirming cell cycle arrest in experiments presented in Fig 4 of the main text. Histograms show DNA content distribution across G0/G1, S, and G2/M phases for each synchronization condition. (TIFF) [file ppat.1013217.s002.tiff]
