# Supplementary material for: Temporal gating of nuclear import: How Merkel cell polyomavirus exploits the cell cycle for nuclear entry
Source: PLoS Pathog. 2025 May 30;21(5):e1013217. doi: 10.1371/journal.ppat.1013217 (PMC12157840; doi:10.1371/journal.ppat.1013217)
Supplement: Attachment — Submitted filename: Response to Reviewers.pdf [file ppat.1013217.s004.pdf]

Reviewer #1:

- Is it possible that the pseudovirus preparations contain both packaged GFP plasmid and unpackaged plasmids that are able to enter the cell and penetrate. Treating the preparations with DNase would eliminate this possibility?

  We have now included a GFP only control (Fig. 1F) to rule out the possibility that the unpackaged plasmids are able to enter the cell and express GFP. We do treat our PsV preparations with DNAse prior to purifying them and have updated the methods section to indicate this.

- A GFP plasmid alone control may shed light on when genome is released from the capsid. Does VP1 enter along with genome or is the genome released first?

  Whether VP1 enters the nucleus along with the genome (such as seen with SV40) is still not clear but is a great question that we are keen to answer. We are currently working to generate BrdU labelled PsV that we can trace through different time points of entry including into the nucleus. Some of our preliminary data shows that VP1 can be observed in the nucleus as early as 24hpi, but we do not know if this is also associated with the "viral genome" i.e. GFP plasmid.

- I suggest using transduction rather than infection given that these are pseudoviruses.

  We can update this if you'd like but is it ok if we just say "PsV infection" since we are using GFP as a readout for viral delivery to the nucleus and not as a method of introducing new genetic material into the cells?

- Also, why are COS7 cells more susceptible to this? Could it have to do with the fact that they express SV40 T antigen that causes the cells to cycle more rapidly?

  This is a great question and one that Reviewer #2 also mentioned. We are not sure, but it is possible that the T antigen makes them more susceptible to MCPyV PsV infection as we produce the PsVs in 293TT cells which also contain SV40 T antigen. We do have a project in our lab that is testing the susceptibility (intracellular VP1) vs permissiveness (GFP expression) across various cell types including human keratinocyte lines in order to answer some of these questions.

Reviewer #2:

- The choice of cell lines to test and the ultimate use of only one cell line throughout this study lessens the impact of these findings. Given that the tropism of the human

MCPyV virus is likely cutaneous, it is intriguing that the authors chose a lung epithelial cell line and monkey kidney fibroblasts for these studies instead of skin-derived keratinocytes and fibroblasts. The use of at least two human cell lines (whether epithelial or fibroblasts) would make these findings more compelling and relevant to the natural tropism of the virus.

We initially used A549 cells for our experiments because an earlier study reported that they were highly susceptible to MCPyV PsV infection (Schowalter, et al., 2012, PMID: 22860078). Since then, they have continued to be used to study the infectious entry pathway of MCPyV in some of the more recent papers on this topic as well (Becker, et al., 2019, PMID: 30626687). We do agree with the reviewer on this point though and have included additional data (Fig. 4H and 4I) to show that the effect of Ro-3306 is consistent across two other cell types. Most relevant to the reviewer comment is the inclusion of non-immortalized normal human dermal fibroblasts. We also attempted to use a K17-expressing keratinocyte cell line called neonatal human telomerase reverse transcriptase (N/TERT) to recapitulate the data, but they were not able to survive 72hr of Ro-3306 treatment to complete the experiments (see below). CV-1 is not a human cell line but does not express SV40 T antigen which suggests that the phenotype we see is not solely dependent on T antigen expression. We hope that the inclusion of this data satisfies the reviewer, and we will continue to search for other human cell lines to include in future studies.

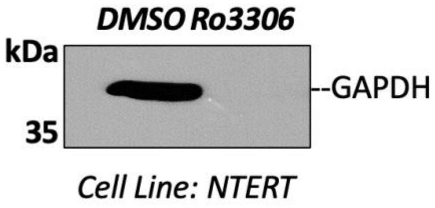

- The use of multiple approaches (pharmacological inhibition with IPZ, siRNA to IPO7, NUP98, and CHMP7, electron microscopy, etc) to interrogate nuclear entry is a strength of this manuscript. The authors interpret their findings that IPZ treatment and NUP98 knockdown significantly reduced infection to mean that a subset of viral particles may still use the NPC for nuclear entry. Given the questions surrounding the role of and necessity for the MCPyV VP1 NLS in nuclear entry, studies using NLS mutagenesis would likely provide opportunities to further clarify the mechanistic role, if any, of the MCPyV VP1 NLS in nuclear entry. Perhaps there is a reason for not doing so (i.e. structural fidelity of capsid). Regardless, the mutagenesis of VP1 NLS would strengthen their findings in addition to drug and KD studies.

We performed mutagenesis to disrupt the putative NLS regions in VP1 (Fig. S1). Unfortunately, this seems to have also disrupted PsV assembly in the nucleus during particle production as we were unable to purify any mutant PsV to test in our

system. However, now that we have this construct, we may be able to use transfected plasmid to further interrogate a role for the VP1 NLS in targeting the PsV to the nuclear membrane through immunoprecipitation studies with nuclear membrane proteins.

- In Figure 5C, there appears to be a mixed population of cells in which a subset has increased membrane permeability following successful CHMP7 knockdown. The use of a GFP reporter allows visual inspection of cells that achieved successful nuclear entry. Could the authors use co-IF to determine if NEBD in cells with CHMP7 knockdown (and cytoplasmic mCherry) is, indeed, coincident with GFP expression on a per-cell basis? Or did they only quantify intracellular GFP in the whole population (Figure 5F)? Such analysis would provide strong evidence (on a per-cell basis) to support their hypothesis that increased nuclear membrane permeability facilitates infection.

  In Fig. 5F, we only quantified intracellular GFP in a whole population. We attempted to perform the suggested experiment by knocking down CHMP7, transfecting mCherry-NLS and infecting with MCPyV-GFP for 48hr, but the cells did not survive 96hr of CHMP7 KD. We also tried to KD CHMP7 and use immunofluorescence microscopy to determine whether this condition leads to increased nuclear VP1 signal as an alternative approach. While we saw a trend towards that direction, it was not statistically significant, so we did not include this data in the revised manuscripts.

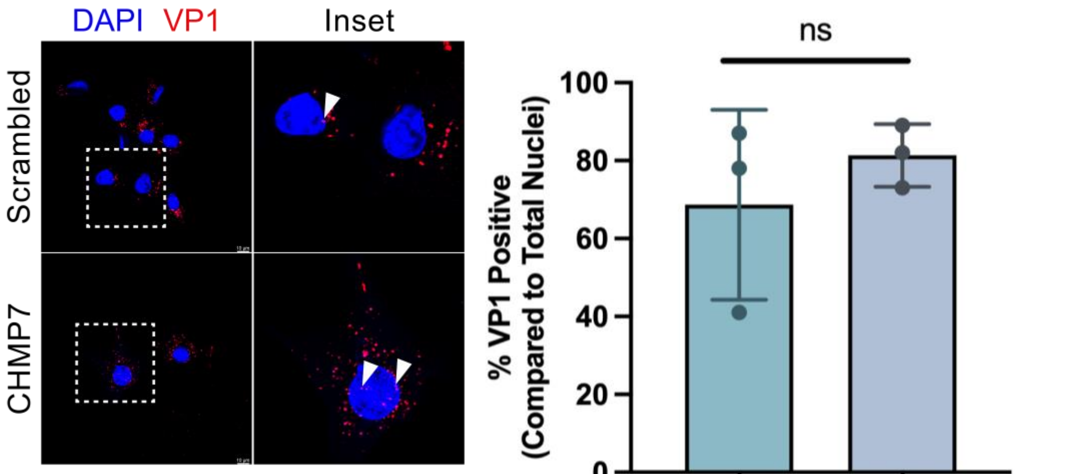

Exp 1.148 MeOh Fixation Only
Nuclear VP1 Detection
Single Plane 48hpi 10uL Virus

- The investigators initially tested three different cell lines for infection with MCPyV PsV (A549, CV-1, and COS-7). Of the three lines tested, COS-7 supported the most robust infection and nuclear entry. Given the current ambiguity surrounding MCPyV tropism, the authors could have leveraged these differences in infectivity to explore

whether cellular tropism is in any way determined by the nuclear entry mechanism they discovered contributes to infectivity. For instance, does CHMP7 knockdown in A549 and CV-1 cells now make them permissive for infection/GFP expression? Is there a qualitative or quantitative difference between epithelial cells (A549) and fibroblasts (CV-1)? Such studies could potentially expand and elevate the impact of these studies.

Thank you for this suggestion. We are working to explore these questions in the lab now by testing the susceptibility of many other cell lines to MCPyV PsV infection. In the future, we will focus on using human cell lines in addition to A549, CV-1 and COS-7 for our studies.

- The header “COS-7 cells support infection” should be more specific. The other cell lines also support ‘infection,’ just at a lower level than COS-7. Also, it seems the authors can only say that cells support PsV infection as this is not a natural MCPyV infection system.

  We have updated the text to reflect this.

- Figure 1C: What are the concentrations of pseudoviruses used for infection? The Materials and Methods section indicates that the PsV stock concentration is determined, but the figure only indicates they are using volume (10-20uL) to infect. Was the percentage of cells that are infected calculated (using GFP immunofluorescence for instance)?

  We use BSA standards and coomassie staining to estimate that our PsV stocks contain 0.5-1ug VP1/mL. We have included this in the materials and methods section. Approximately 5% of cells well express GFP when infected by 10uL of MCPyV PsV as determined by both flow cytometry and immunofluorescence microscopy for GFP expression.

- In Figure 4, was activity of their inhibitors of cell cycle progression (aphidocolin and RO-3306) confirmed to affect the cell cycle as predicted in each experiment? Or was it only performed in the independent experiment shown in Figure 4B? Given the extent to which they are interpreting these results, it would be useful to include these controls in every experiment, either by flow cytometry or some other biomarkers of cell cycle stage, to validate the inhibitors are functioning as expected.

  Yes, we confirmed that the inhibitors affect the cell cycle as expected for each experiment and have included this information for each experiment in Fig. 4 and have included this data in the supplementary materials (Fig. S2).

- Materials & Methods
  a. siRNA sequences for CHMP7 and IPO7 need to be included.

  We have now included this information.

  b. Do specific catalog numbers and application-specific dilutions for antibodies need to be listed?

  We have included the catalog numbers for the antibodies.

  c. Need to add methods sections for Western blotting (how were lysates made, membrane, protein concentration loaded, transfer buffer, etc) and PsV infection (are cells prepared for infection, is a specific media used, is viral inoculum replaced or left on cells, etc).

  We have updated this information as well.

Reviewer #3:

- Dharmacon should provide SMARTpool siRNA sequences for publication if you reach out to them and these should be included in the methods.

  Thank you, we have now included these sequences in the materials and methods section.

- There is some concern about quantifications of the westerns from film as the methods are not clearly stated whether HRP is used, which may not provide for a quantitative analysis of protein levels over what appears to be a broad range. Secondary antibodies were not mentioned.

  We have added a section on western blot quantification to clarify this point.

- The authors also need to report the efficiency of the real-time qPCR reactions.

  We have included this in the materials and methods section called "Quantification of intracellular GFP".

- In figure legend 1E (lines 466-467) the authors need to clarify if they are quantifying the GFP reporter mRNA or DNA plasmid.

  We have now clarified that we are referring to DNA plasmid in the figure legend.

- The authors need to make it clear in the figure legends whether pretreatment with drug extends past and during viral infection, see figure 2.

  We have added the duration of drug treatment to the legend and materials and methods sections.

- In some of the figures the labels are too small and would be easier to read if they were larger fonts. Also, the fonts between the westerns and the graphs are not the same. Examples are figure 4 graphs and experimental diagrams, 2, 5D, 5F, 3D, 3F and 1E. Probably removing the bold font and increasing the font size would help a lot.

  Thank you for this feedback. We have updated this to improve accessibility.
